# Periapical Bone Healing of a Large Radicular Cyst Following Surgical Endodontic Treatment Without Regenerative Procedures: A 2.5‐Year Follow‐Up Case Report

**DOI:** 10.1002/ccr3.71791

**Published:** 2025-12-29

**Authors:** Anisha Mishra, Jitendra Sharan, Anand Marya

**Affiliations:** ^1^ Department of Dentistry All India Institute of Medical Sciences Bhubaneswar Odisha India; ^2^ Faculty of Dentistry University of Puthisastra Phnom Penh Cambodia

**Keywords:** apicoectomy, cyst, periapical lesion, regeneration, self‐healing

## Abstract

This case report highlights the conservative management of a large peri‐apical lesion involving multiple teeth of the maxilla where the lesion eroded the maxilla's palatal, buccal, and nasal plates with a mobile central incisor. Affected teeth were splinted and treated with root canal treatment, followed by a surgical approach to remove the cystic lining and retrograde filling with MTA. No regenerative graft or scaffold was placed on the bony defect, and the patient was followed regularly. Over two and a half years, the cystic lesion showed positive signs of healing with new bone formation, thereby reducing the cystic space; the teeth were asymptomatic, and the mobile incisor became stable. The lesion volume decreased 77% from 2.951 to 0.682 cm^3^. This shows that in young patients with economic constraints and in facilities with very limited feasibility for regenerative techniques, proper non‐surgical endodontic treatment followed by enucleation of the cyst with periodic review calls can aid in saving teeth with a compromised prognosis.

## Key Clinical Message

Clinicians should attempt to conserve teeth with guarded/poor prognosis prior to extraction in young, healthy individuals with economic constraints and in facilities with no feasibility for regenerative techniques. This case report highlights self‐healing a large through‐and‐through lesion over 2.5 years, by 77% without the use of any regenerative technique.

## Graphical abstract

Comprehensive surgical management of a large radicular cyst without regenerative protocols.

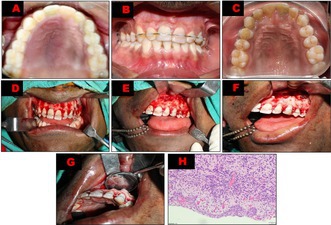

## 1 Introduction

Traumatic dental injuries can lead to several complications, with pulpal and periodontal involvement strongly influencing the long‐term outcome [1, 2]. When the vascular supply or pulpal vitality is compromised, necrosis may occur, and microbial contamination of the necrotic pulp can subsequently lead to periapical pathology [3, 4, 5]. Conventional endodontic therapy aims to control infection, resolve inflammation, and create conditions favorable for tissue repair [6]. However, the size of the preoperative lesion remains one of the most important predictors of healing, and large periapical lesions are particularly challenging to manage [7].

In extensive defects, fibroblastic ingrowth from the periosteum may result in scar tissue rather than true bone regeneration, potentially compromising long‐term success [8, 9]. Treatment options range from non‐surgical root canal therapy to periapical surgery or extraction, with surgical management demonstrating success rates approaching 90% when combined with modern microsurgical techniques and biocompatible root‐end materials [6, 10, 11]. Regenerative procedures, including grafts and membranes, may further enhance healing in large defects by providing scaffolds for osteogenesis [12].

Despite these advances, access to regenerative materials is not universal, and evidence showing predictable spontaneous bone healing in large lesions remains limited. This case report highlights the successful resolution of a large radicular cyst using conservative and surgical endodontic treatment without regenerative adjuncts, demonstrating an important clinical alternative in settings with economic or logistical constraints. The report follows the Preferred Reporting Items for Case Reports in Endodontics (PRICE) 2020 guidelines [13].

## 2 Clinical Case Presentation

A 23‐year‐old male patient visited the dental OPD with a chief complaint of swelling in the roof of his mouth for one and a half months. The swelling had increased in size and was associated with localized, needle‐pricking type of pain, which used to get aggravated on biting. The patient gave no relevant medical or past dental history. The patient provided no past history of trauma, hit, or fall. The patient also gave no history of smoking/tobacco or any related addiction habits.

**FIGURE 1:**
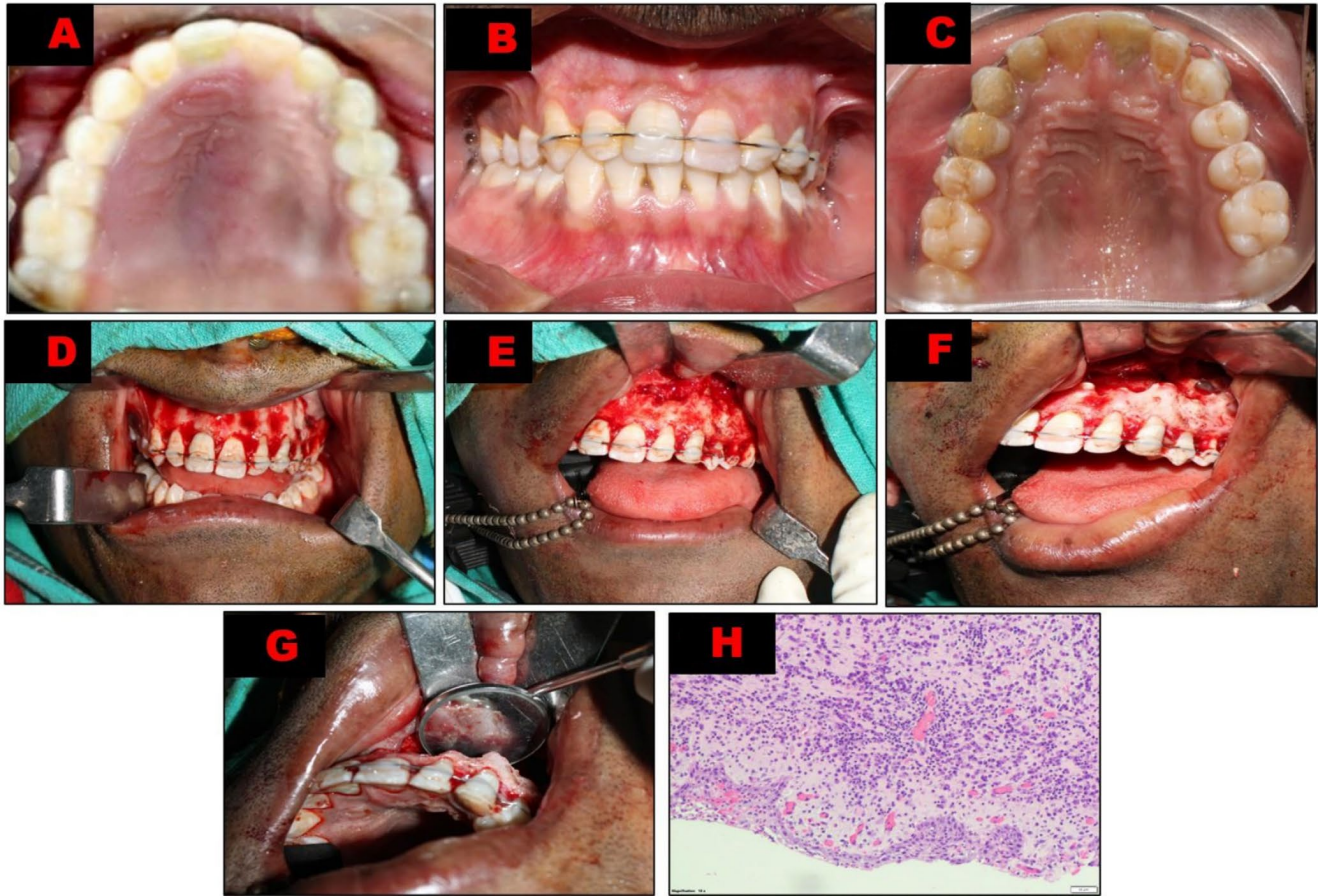
Clinical and Histopathology images. (A) Pre‐operative occlusal view showing palatal swelling. (B) Frontal view after 0.016″ round stainless wire splint placement. (C) Occlusal view at 2.5 years follow up showing complete healing of the palatal swelling. (D) Intra‐operative surgical image showing two vertical releases and one horizontal incision extending from a distal aspect of 12 to a distal aspect of 25 using a microblade. (E) Intra‐operative surgical image showing flap raised and retracted with periosteal elevators and an Austin retractor to expose the bony crypt created using a high‐speed surgical handpiece. (F) Intra‐operative surgical image showing 3 mm resection of all the affected root tips. (G) Intra‐operative surgical image showing retrograde MTA filling into the resected roots. (H) Histopathological image of the specimen.

On extraoral examination, the face appeared apparently bilaterally symmetrical with no signs of swelling, sinus tract, or fistula. Intraoral examination revealed discolored 21 with Miller's grade II mobility with no tenderness on vertical and horizontal percussion. Thermal and electric pulp testing (Parkel Electronics Division, Farmingdale, New York, USA) elicited a negative response for 21 and a heightened response even after stimuli retrieval in relation to 11, 22, 23 and 24.11, 22, 23 and 24 showed mild tenderness on vertical and horizontal percussion. The gingival attachment apparatus was apparently normal. A palatal swelling with a smooth surface size 4 × 3 × 2 cm^3^ was evident, extending to palatal gingival margins of 11, 21, 22, 23, 24 (Figure 1A). The swelling was round in shape, soft, non‐fluctuant in consistency, pale pink in color, mildly tender on palpation, and was not fixed to the underlying tissues.

**FIGURE 2:**
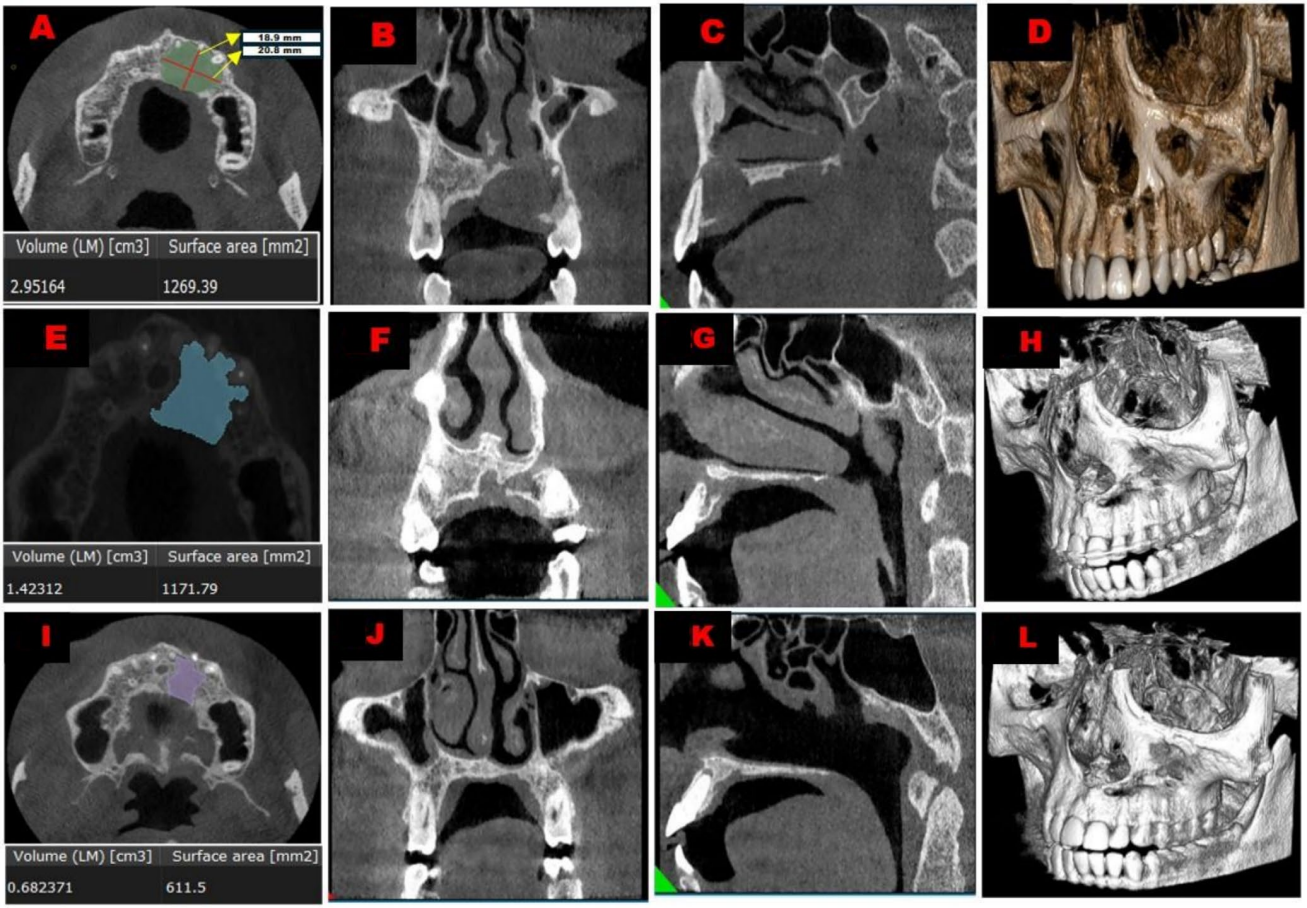
Cone beam computed tomography (CBCT) images. (A) Pre‐operative axial view of the volumetric and surface area analysis along with the maximum diameter of the cystic lesion. (B) Pre‐Operative coronal view of lesion infiltrating the floor of the nasal cavity. (C) Pre‐Operative sagittal view of lesion infiltrating the floor of the nasal cavity. (D) Pre‐operative 3‐Dimensional model showing loss of buccal cortical plate and nasal floor. (E) Axial View of volumetric and surface area analysis of the cystic lesion at 6 months follow up. (F) Coronal view of the lesion at 6 months follow up showing signs of bone formation. (G) Sagittal view of the lesion at 6 months follow up showing signs of bone formation. (H) 3‐Dimensional model of the lesion at 6 months follow up showing signs of bone formation. (I) Axial View of volumetric and surface area analysis of the cystic lesion at 2.5 years follow up. (J) Coronal view of the lesion at 2.5 years follow up showing further signs of bone formation. (K) Sagittal view of the lesion at 2.5 years follow up showing further signs of bone formation. (L) 3‐Dimensional model of the lesion at 2.5 years follow up showing further signs of bone formation.

A cone beam computed tomography (CBCT) scan was done, which showed a fairly large cyst in relation to the root apices of 11, 21, 22, 23, 24 with perforation of the palatal, buccal cortical plate and superiorly infiltrating the nasal floor with maximum diameter size of 1.89 × 2.08 cm when measured in the axial section, and the volume of the lesion was 2.951 cm^3^ (Figure 2A–D). CBCT also revealed vertical bone loss up to the middle‐apical third of the root and a complete pulp canal obliteration in relation to 21. A diagnosis of pulp necrosis with a chronic periapical abscess in relation to 21 and asymptomatic irreversible pulpitis with a chronic periapical abscess in relation to 11, 22, 23 and 24 was given. After discussion of the case with the maxillofacial surgeons and patient, the treatment option of splinting of upper anterior teeth, non‐surgical endodontic treatment of 11, 22, 23, 24 followed by surgical endodontic treatment, that is, apicoectomy of the affected teeth was put forth, and the patient accepted the same. The need for bone grafting to aid in speedy and enhanced healing of the large cystic space was explained to the patient, but the patient did not accept this option due to monetary constraints.

## 3 Treatment Procedure

### 3.1 Non‐Surgical Procedures

Preoperative explanations of the benefits and risks associated with surgical and non‐surgical endodontic procedures, such as bleeding, swelling, pain, mobility, gingival recession, and vertical root fracture, were discussed with the patient. A verbal and written consent form was obtained from the patient. Preoperative blood tests were taken, including complete blood count, prothrombin time, activated partial thromboplastin time, serology (HIV, HCV, and HBsAg), and International Normalized Ratio (INR), and the values were all within normal parameters.

Pre‐treatment impression of the upper arch was made with irreversible alginate (Tropicalgin, Zhermack S.P.A, Italy), and a 0.016″ round stainless wire (G & H Orthodontics, IN, USA) was adapted from upper right canine to left second premolar. Later, it was bonded onto the labial surfaces of the teeth with a flowable composite (Tetric PowerFlow, Ivoclar Vivadent, Schaan, Liechtenstein) (Figure 1B).

Non‐surgical endodontic treatment was initiated after anesthetizing 11, 22, 23, 24 using three cartridges of 1.8 mL of 2% lidocaine containing 1:200,000 epinephrine. Rubber dam (Coltene Whaledent Inc., Ohio, USA) isolation was done and access opening was initiated using an endo access bur (#1) at high speed (Dentsply Sirona, Tulsa, USA). Working length was determined using an electronic apex locator (Root ZX; Morita, Tokyo, Japan) and confirmed with RadioVisioGraphy. ProTaper Gold files (Dentsply Maillefer, Ballaigues, Switzerland) were used for shaping and cleaning by the crown‐down technique. The instrumentation was performed using 2.5% sodium hypochlorite solution (Sodium Hypochlorite, Chemident, India) and normal saline. Final irrigation was performed with 2.5% sodium hypochlorite solution, 17% EDTA (MD‐Cleanser, Meta‐Biomed, Republic of Korea), and normal saline (Otsuka Pharmaceuticals, India). The canals were medicated with Calcium hydroxide paste (ApexCal, Ivoclar Vivadent, Schaan, Liechtenstein) using a lentulo spiral (Dentsply Maillefer, Ballaigues, Switzerland), and the access cavity was sealed with Cavit (3 M ESPE Dental Products, St Paul, MN, USA). The patient was prescribed antibiotics and analgesics [Amoxycillin (500 mg) + Clavulanic Acid (125 mg)] twice a day for 5 days and [Aceclofenac (100 mg) + Paracetamol (325 mg) on an SOS basis] following the endodontic procedure. The patient was asymptomatic during recall after 2 weeks. Calcium hydroxide was removed, and obturation was done using the lateral condensation technique in the anterior teeth and the single cone obturation technique in the premolar. The access cavities were then sealed using resin composite (Z‐100; 3 M ESPE Dental Products, St Paul, MN, USA).

### 3.2 Surgical Endodontic Procedure

One week later, the patient was scheduled for the apicoectomy procedure and was advised to take analgesic tablets before the surgery. The patient was instructed to rinse the oral cavity with chlorhexidine gluconate 0.2% w/v solution (Clohex mouth‐wash, Dr. Reddy's laboratories, India). This was followed by cleaning the surgical site with a Povidone‐Iodine solution of 10% w/v (Win‐Medicare, India). Probing depth at the surgical site ranged between 1 and 2 mm; the gingival biotype was thick, and a medium upper smile line was present. Three cartridges of 1.8 mL of 2% lidocaine containing 1:200,000 epinephrine were administered for infraorbital and nasopalatine nerve blocks. Two more cartridges were injected for buccal and palatal infiltrations for further localized anesthesia. After achieving complete anesthesia confirmed by subjective signs, a rectangular papillary‐based flap was raised with two vertical releases and one horizontal incision extending from a distal aspect of 12 to a distal aspect of 25 using a microblade (Figure 1D). The flap was then raised and retracted with periosteal elevators and an Austin retractor. Round bur No. 6 and No. 701 bur were used to create the bony crypt in the labial aspect of the teeth using a high‐speed surgical handpiece (Figure 1E). Saline irrigation was continuously done to reduce the frictional heat during the osteotomy procedure. Enucleation of the cyst and other apical soft tissue was excised using surgical curettes until the root tips were evident. The specimen was immersed in buffered formalin and sent for histopathological examination. Cotton pellets soaked in local anesthesia (2% lidocaine containing 1:200,000 epinephrine) were placed into the crypt to achieve hemostasis. The apical 3 mm of all the teeth were resected at 90° to the long axis of the tooth using a round bur (Figure 1F). Furthermore, 3 mm of apical cavities were prepared into all the teeth using ultrasonic root‐end tips (KiS‐2D tip), and a retrograde filling of mineral‐trioxide‐aggregate (ProRoot‐MTA) was condensed and placed using micro pluggers into the cavities (Figure 1G).

The reflected flap was irrigated, moistened with normal saline, and repositioned with gentle pressure. Seven interrupted sutures using braided coated polyglactin 3‐0 sutures (Vicryl Plus, Ethicon LLC, USA) were placed using a Castro Viejo needle holder and scissors. The tissue was again cleaned with betadine and gently compressed with saline for 2 min. A postoperative periapical radiograph showed satisfactory root‐end resection and retrograde plugs. Before discharge, the patient was prescribed antibiotics and analgesics (Amoxycillin (500 mg) + Clavulanic Acid (125 mg)) twice a day for 5 days and (Aceclofenac (100 mg) + Paracetamol (325 mg)) for 3 days, and post‐surgical instructions regarding the care of the surgical site and maintenance of optimal oral hygiene were given.

## 4 Histopathology Report

Multiple fragments of tissue focally lined by ulcerated epithelium were evident. The sub‐epithelium showed granulation tissue formation. Dense inflammatory infiltrates comprising predominantly plasma cells and neutrophils were evident. Surrounding areas showed fibroblastic proliferation with numerous capillaries and perivascular moderate plasmocytic inflammatory infiltrates, which was a confirmatory diagnosis of radicular cyst (Figure 1H).

## 5 Postoperative Review

The patient was recalled after 1 week post‐surgically. Clinical assessment showed the patient had mild swelling and no discomfort. Soft tissue healing was satisfactory. The importance of maintaining good oral hygiene was reemphasized. At the six‐month follow‐up, the stabilizing splint was removed after the radiographic and clinical evaluation, indicating no further signs of mobility in relation to tooth 21 (Figure 2E–G). The patient was subsequently reviewed at 1, 1.5, 2 and 2.5 years later and was completely asymptomatic in every visit. The lesion volume had decreased by 77% from 2.951 to 0.682 cm^3^ (Figure 2A,E,H,I) at the 2.5‐year review. Healing was satisfactory clinically and radiographically (Figures 1C and 2I–L).

## 6 Patient Experience and Compliance

Throughout the follow‐up period, the patient reported a consistently positive experience, with minimal postoperative discomfort and no functional limitations after the initial healing phase. He demonstrated excellent compliance with oral hygiene measures, medication instructions, and scheduled reviews, which contributed meaningfully to the favorable healing progression. By the six‐month follow‐up, the patient expressed satisfaction with the resolution of symptoms, restoration of comfort during mastication, and the return of normal esthetics. At subsequent visits up to 2.5 years, he remained symptom‐free and highlighted the psychological reassurance gained from retaining his natural teeth despite the initial severity of the lesion. These patient‐reported outcomes reinforce the clinical viability of conservative surgical management in young, motivated individuals.

## 7 Discussion

Periapical lesions most commonly arise from microbial penetration into the root canal system; however, in the present case, the absence of caries and the presence of a discolored, mobile central incisor indicate trauma‐induced pulpal necrosis as the initiating factor. Chronic inflammation stimulates epithelial rests of Malassez, leading to cyst formation and enlargement through IL‐17, M1 macrophage recruitment, and RANKL‐mediated osteoclastic pathways, mechanisms known to progressively thin surrounding cortical plates [14, 15].

Although guided tissue regeneration is often recommended for large defects, spontaneous bone regeneration remains a predictable outcome when periosteal integrity and vascularity are preserved. The periosteum's cambium layer provides osteoprogenitor cells, angiogenic support, and growth factors essential for intramembranous ossification once the inflammatory source is removed [16]. Studies have demonstrated that significant healing can occur without biomaterials, with large defects showing progressive mineralization over time [17, 18, 19]. Similar results have also been observed in maxillary cystic lesions, where substantial spontaneous bone fill occurred even in the absence of grafting materials [20].

Notably, Chiapasco et al. reported uneventful spontaneous bone regeneration in all cases of large mandibular cysts treated without grafting, with the physiologic organization of the blood clot alone being sufficient for new bone formation [19]. Their analysis further emphasized that autogenous bone grafts can increase postoperative morbidity including infection, graft resorption, and donor‐site complications while allogeneic and xenogeneic materials lack intrinsic osteogenic potential and may prolong healing due to slow graft substitution. Thus, when biological conditions are favorable, avoiding grafts does not compromise healing outcomes.

Patient‐related variables also significantly influence regenerative potential. Younger individuals exhibit greater periosteal cellularity and osteogenic capacity, whereas systemic factors such as metabolic disorders, smoking, or medications affecting bone turnover may delay healing. Lesion type and morphology further modulate outcomes; radicular cysts generally heal well once the inflammatory stimulus is eliminated, although large perforations or through‐and‐through defects may slow regeneration due to reduced bony containment [17, 19].

In the present case, despite the lesion's size and the absence of grafts or membranes due to feasibility constraints, follow‐up at 2.5 years demonstrated substantial spontaneous healing, with increased radiopacity, approximately 77% reduction in cyst volume, and complete stabilization of the involved tooth. These findings align with evidence that periosteum‐driven osteogenesis can effectively restore even large periapical defects when infection is controlled and biological conditions are favorable [17, 18, 19, 20].

## 8 Conclusion

In young, healthy individuals with economic constraints and in facilities with no feasibility for regenerative techniques, an attempt must be made to conserve the affected teeth with a guarded/poor prognosis prior to extraction. This case report highlights the beauty of self‐healing a large through‐and‐through lesion by 77% without the use of any regenerative technique.

## Author Contributions

**Anisha Mishra:** conceptualization, data curation, formal analysis, investigation, methodology, supervision, validation, visualization, writing – original draft, writing – review and editing. **Jitendra Sharan:** conceptualization, formal analysis, funding acquisition, investigation, methodology, supervision, validation, visualization, writing – original draft, writing – review and editing. **Anand Marya:** conceptualization, funding acquisition, resources, software, writing – original draft, writing – review and editing.

## Funding

The authors have nothing to report.

## Ethics Statement

This case report was exempt from formal ethical approval, as it involves a single patient with anonymized data; written informed consent was obtained from the patient for publication, including the use of clinical images.

## Conflicts of Interest

The authors declare no conflicts of interest.

## Data Availability

Datasets related to this article will be available upon request to the corresponding author.
